# A Randomized, Double‐Blind, Placebo‐Controlled, Phase 1 Study to Evaluate the Safety, Reactogenicity, and Immunogenicity of Single Vaccination of Ad26.RSV.preF‐Based Regimen in Japanese Adults Aged 60 Years and Older

**DOI:** 10.1111/irv.13336

**Published:** 2024-06-16

**Authors:** Takashi Eto, Yusuke Okubo, Atsushi Momose, Hiroshi Tamura, Richuan Zheng, Benoit Callendret, Arangassery Rosemary Bastian, Christy A. Comeaux

**Affiliations:** ^1^ SOUSEIKAI Hakata Clinic Fukuoka Japan; ^2^ Janssen Pharmaceutical K.K. Tokyo Japan; ^3^ Janssen Vaccines & Prevention B.V. Leiden The Netherlands

**Keywords:** adenovirus serotype 26, respiratory syncytial, respiratory syncytial virus vaccine, virus fusion proteins, virus adult vaccination

## Abstract

**Background:**

Respiratory syncytial virus (RSV) is increasingly recognized as a significant cause of lower respiratory tract disease (LRTD) in older adults. The Ad26.RSV.preF/RSV preF protein vaccine demonstrated protective efficacy against RSV related LRTD in a Phase 2b study in the United States. Hence, Ad26.RSV.preF/RSV preF protein vaccine candidate was evaluated in the Japanese older adult population.

**Methods:**

This Phase 1 study evaluated safety, reactogenicity, and immunogenicity of Ad26.RSV.preF/RSV preF protein vaccine at dose level of 1 × 10^11^ vp/150 μg in Japanese healthy adult aged ≥60 years. The study included a screening Phase, vaccination, 28‐day follow up Phase, a 182‐day follow‐up period, and final visit on Day 183. A total of 36 participants were randomized in a 2:1 ratio to receive Ad26.RSV.preF/RSV preF protein vaccine (*n* = 24) or placebo (*n* = 12). After study intervention administration, the safety and immunogenicity analysis were performed as per planned schedule. Immune responses including virus‐neutralizing and preF‐specific binding antibodies were measured on Days 1, 15, 29, and 183.

**Results:**

There were no deaths, SAEs, or AEs leading to discontinuation reported during the study. The Ad26.RSV.preF/RSV preF protein vaccine had acceptable safety and tolerability profile with no safety concern in Japanese older adults. The Ad26.RSV.preF/RSV preF protein vaccine induced RSV‐specific humoral immunity, with increase in antibody titers on Days 15 and 29 compared with baseline which was well maintained until Day 183.

**Conclusions:**

A single dose of Ad26.RSV.preF/RSV preF protein vaccine had an acceptable safety and tolerability profile and induced RSV‐specific humoral immunity in Japanese healthy adults.

**Trial Registration:**

NCT number: NCT04354480; Clinical Registry number: CR108768.

## 1 Introduction

Human respiratory syncytial virus (RSV) is a non‐segmented RNA virus from the Pneumovirus family, with two major antigenic subtypes, namely, RSV‐A and RSV‐B. RSV is an important cause of serious respiratory infections in adults aged ≥ 60 years, immunocompromised, and those with underlying chronic cardiopulmonary conditions [1]. Although almost all children have been infected with RSV by the age of 2 years, incomplete and short‐lived natural immunity leads to repeated RSV infections throughout life [2, 3, 4, 5]. Older adults may experience moderate to severe disease after RSV infection causing significant morbidity and mortality. In long‐term care facilities, RSV is estimated to infect 5%–10% of the residents each year with significant rates of pneumonia (10%–20%) and death (2%–5%) [6]. In recent systemic literature review of RSV burden, it was estimated that the proportion of RSV‐positive cases among older adults varied from 5.09% in Europe to 4.49 in North America, to 3.45 in the Western Pacific region, annually. The same study also reported that 27.44% of RSV patients developed pneumonia, 24.48% required hospitalization, and 5.01% were admitted to the ICU. Case fatality proportion (CFP) among older adults was 8.18% [7]. A multicenter prospective observational cohort study conducted in Japan during the 2019–2020 season (before COVID‐19) reported that RSV was a major pathogen for respiratory infections in older adults with 2.4% occurrence rate of RSV–acute respiratory disease (ARD) [8]. This high disease burden highlights the need to evaluate prophylactic approaches against RSV in older adults [9].

The investigational vaccine that we evaluated in the trial reported here is the combination of an adenovirus serotype 26 RSV vector encoding RSV F protein stabilized in the conformation prefusion (preF) (Ad26.RSV.preF) and an RSV preF protein. In preclinical studies, adenovirus serotypes 26 (Ad26) expressing F protein of the RSV induced strong RSV‐specific humoral and cellular immune responses [10, 11]. The addition of RSV preF protein to Ad26.RSV.preF improved humoral responses while maintaining cellular responses and provided protection from RSV‐A2 challenge superior to that provided by either component alone [12]. In humans, a single Ad26.RSV.preF immunization was immunogenic, had an acceptable safety profile, and demonstrated the ability to substantially reduce RSV infection and disease in a human challenge study [13, 14]. The immunological benefit of combining Ad26.RSV.preF with RSV preF protein was shown in a Phase 1/2a study where a significant increase in virus‐neutralizing titers was observed after 28 days post dose 1 in the groups combining Ad26.RSV.preF and RSV preF protein compared to Ad26.RSV.preF alone [15]. Furthermore, in Phase 2b study, Ad26.RSV.preF/RSV preF protein vaccine candidate at dose level of 1 × 10^11^ vp/150 μg was highly efficacious against RSV‐mediated lower respiratory tract disease, with an acceptable safety profile in adults aged ≥ 65 years [16].

Here, we report the results of Phase 1 study conducted to evaluate safety, reactogenicity, and immunogenicity of Ad26.RSV.preF/RSV preF protein vaccine at dose level of 1 × 10^11^ vp/150 μg in Japanese healthy participants aged ≥ 60 years.

## 2 Materials and Methods

### 2.1 Study Design

This was a single‐center, randomized, placebo‐controlled, double‐blind Phase 1 study in Japanese adults aged ≥ 60 years who were in stable health to evaluate safety, reactogenicity, and immunogenicity of Ad26.RSV.preF/RSV preF protein vaccine at dose level of 1 × 10^11^ vp/150 μg. The trial protocol, including amendments, was approved by the ethics committee at the participating center. The trial was conducted in accordance with the Declaration of Helsinki and principles of Good Clinical Practice. All participants provided written informed consent.

### 2.2 Study Participants

Healthy or medically stable, ≥ 60‐year‐old Japanese participants were included in the study.

### 2.3 Eligibility Criteria

Participants with underlying illnesses such as hypertension, Type 2 diabetes mellitus, hyperlipoproteinemia, or hypothyroidism who had to be medically controlled or under treatment with a stable dose of medication for at least 12 weeks preceding vaccination and the dose had to remain stable for the duration of the study were included.

Participants who had significantly acute illness or temperature ≥ 38.0°C within 24 h prior to the planned dose of study intervention; received treatment with immunoglobulins in the 2 months, immunoglobulins specific to RSV, human metapneumovirus or parainfluenza viruses in the last 12 months, or blood products in the 4 months before the planned administration of the study intervention or had any plans to receive such treatment during the study; and who had a history of malignancy within 5 years before screening were excluded. Participants with serious chronic disorder, including severe chronic obstructive pulmonary disease or clinically significant congestive heart failure, end‐stage renal disease with or without dialysis, clinically unstable cardiac disease, and Alzheimer's disease, were also excluded.

### 2.4 Study Procedure and Assessments

The study included a screening phase (Day −28 to Day −1), vaccination (Day 1), a 28‐day follow up phase (including hospitalization phase from Day 1 to Day 2, outpatient visit on Day 3, home assessment phase from Days 4 to 7, and outpatient visits on Days 8, 15, and 29), a 182‐day follow‐up period after the vaccination, and a final visit on Day 183. The study duration was approximately 30 weeks per participant. During screening, participants were evaluated for study entry based on the study inclusion/exclusion criteria. A total of 36 participants were planned to be enrolled and randomized in a 2:1 ratio to one of the two study intervention groups to receive a single intramuscular (IM) injection of the Ad26.RSV.preF/RSV preF protein vaccine (1 × 10^11^ vp/150 μg) or placebo. Placebo was normal saline solution.

Postvaccination, participants were closely observed to monitor for the development of any acute reactions for a minimum of 30 min or longer if deemed necessary by the investigator. Any unsolicited, solicited local or systemic adverse events (AEs) were documented by study‐site personnel following this observation period. Participants were provided with a thermometer, a ruler, and a participant diary and were instructed to measure and record solicited local and systemic AEs and body temperature (beginning on the evening of Day 1) daily for 7 days' postvaccination. Participants were followed up until Day 183 (solicited AEs for initial 7 days, unsolicited AEs for until Day 29 and serious AEs [SAEs] for until Day 183).

The specific immunogenicity and safety variables were assessed during the study. The safety assessments were based on AEs (unsolicited, solicited local or systemic AEs; serious AEs), physical examinations, vital sign measurements, electrocardiogram (ECGs), and clinical laboratory tests (blood chemistry and hematology). Any clinically significant abnormalities persisting at the end of the study/early withdrawal were followed by the investigator until resolution or until a clinically stable endpoint was reached. Participants were also asked to record symptoms of the following AEs:

- Solicited local AEs: injection site pain/tenderness, erythema, and swelling at the injection site. The extent (largest diameter) of any erythema and swelling were measured (using the ruler supplied).
- Solicited systemic AEs: fatigue, headache, nausea, myalgia, and fever.

Immunogenicity assessments were performed on Days 1, 15, 29, and 183. Venous blood samples of approximately 8.5 and 30 mL were collected for assessment of humoral and cellular immune responses, respectively. Neutralizing antibodies to vaccine strain (RSV neutralization A), antibodies binding to RSV F protein in pre‐fusion and/or post‐fusion form, cross‐neutralizing antibodies to B and/or a different A strain (RSV strain cross‐neutralization), and neutralizing antibodies to adenovirus (adenovirus neutralization assay) were analyzed (F protein antibody) to evaluate humoral immune response. RSV‐A and RSV‐B neutralizing antibodies were measured with the use of virus‐neutralization assays. RSV F proteins in pre‐fusion and/or post‐fusion form were measured with an enzyme‐linked immunosorbent assay [17].

### 2.5 Statistical Analysis

No statistical hypothesis testing was planned for this study. The study evaluated whether a Ad26.RSV.preF/RSV preF protein vaccine was safe, well tolerated, and immunogenic in Japanese adults aged ≥ 60 years. Primary analysis was performed at 28 days' postvaccination where safety, reactogenicity, and immunogenicity (if data are available) were assessed in all participants. This analysis was performed when all participants had finished their 28‐day post study vaccination visit or had discontinued earlier to evaluate safety after a single dose of Ad26.RSV.preF/RSV preF protein vaccine.

Final analysis was performed at study end, the point when all participants had reached their final visit at Day 183 or discontinued earlier. The analysis sets included full analysis set (FAS) and per‐protocol immunogenicity (PPI) set. Participants with major protocol deviations expecting the impact on immunogenicity outcomes were excluded from PPI set.

The FAS was used for safety evaluations, including all randomized and vaccinated participants, regardless of the occurrence of protocol deviations. All reported AEs with onset after vaccination were included in the analysis. For each AE, the number and percentage of participants who experienced at least one occurrence of the given event were summarized by group. Solicited local (at injection sites) and solicited systemic AEs were summarized descriptively. The overall frequencies per intervention group as well as frequencies according to severity and duration were calculated for solicited AEs. Frequencies of unsolicited AEs were presented by system organ class (SOC) and preferred term (PT). Clinical laboratory, physical examination, and vital signs abnormalities emerging after vaccination were tabulated by the worst abnormality grade.

PPI set was used for immunogenicity analysis. The immunogenicity analysis was done by intervention groups. Descriptive statistics (geometric mean [GM] and 95% confidence interval [CI]) for enzyme‐linked immunosorbent assay (ELISA) and RSV neutralization assay was calculated for continuous immunologic parameters at all time points. GM fold rise from baseline and corresponding 95% CIs were additionally calculated.

## 3 Results

A total of 36 participants were randomized in the study: 24 participants in the Ad26.RSV.preF/RSV preF protein intervention group and 12 participants in the placebo group. All participants completed the study (Table S1). Of the 36 participants enrolled, 20 (55.6%) were men, and 16 (44.4%) were women. The median age of the participants was 64.5 (range: 60–79) years, and the median body mass index was 23.15 (range: 17.8–29.3) kg/m^2^. Demographics and baseline characteristics were comparable among the intervention groups (Table 1).

**TABLE 1:** Summary of demographics and baseline characteristics.

	Ad26/protein preF RSV vaccine	Placebo	All participants
Analysis set: full analysis set	24	12	36
Age, years			
*N*	24	12	36
Mean (SD)	66.0 (4.64)	65.3 (4.11)	65.7 (4.42)
Median	65.5	64.0	64.5
(Min, max)	(60; 79)	(61; 73)	(60; 79)
60–64	11 (45.8%)	7 (58.3%)	18 (50.0%)
65–74	12 (50.0%)	5 (41.7%)	17 (47.2%)
75–84	1 (4.2%)	0	1 (2.8%)
> = 85	0	0	0
Sex			
*N*	24	12	36
Female	10 (41.7%)	6 (50.0%)	16 (44.4%)
Male	14 (58.3%)	6 (50.0%)	20 (55.6%)
Race			
*N*	24	12	36
Asian	24 (100.0%)	12 (100.0%)	36 (100.0%)
Weight, kg			
*N*	24	12	36
Mean (SD)	61.44 (11.144)	61.26 (9.814)	61.38 (10.577)
Median	60.90	62.25	62.15
(Min, max)	(42.2; 82.2)	(43.5; 77.0)	(42.2; 82.2)
Height, cm			
*N*	24	12	36
Mean (SD)	163.38 (8.434)	161.67 (8.780)	162.81 (8.464)
Median	165.25	162.65	165.25
(Min, max)	(145.0; 179.2)	(149.3; 172.2)	(145.0; 179.2)
Body mass index, kg/m^2^			
*N*	24	12	36
Mean (SD)	22.93 (3.245)	23.33 (2.258)	23.06 (2.926)
Median	22.55	23.75	23.15
(Min, max)	(17.8; 29.3)	(19.5; 26.0)	(17.8; 29.3)

*Note: N* for each parameter reflect non‐missing values.

Abbreviations: *N* = total number of patients, SD = standard deviation.

### 3.1 Safety

The Ad26.RSV.preF/RSV preF protein vaccine had an acceptable safety and tolerability profile in Japanese adults aged ≥ 60 years who were in stable health with no safety concern identified during the study. There were no deaths, SAEs, or AEs leading to discontinuation reported during the study. None of the participants reported Grade ≥ 3 AEs (solicited or unsolicited) except headache (solicited systemic, Grade 3) reported by one participant who received Ad26.RSV.preF/RSV preF protein vaccine (Table 2).

**TABLE 2:** Adverse events summary.

	Ad26/protein preF RSV vaccine (*N* = 24)	Placebo (*N* = 12)
Any AE leading to discontinuation of the study	0	0
Any serious AE	0	0
Any solicited AE, *n* (%)	19 (79.2%)	5 (41.7%)
Any at least Grade 3 solicited AE, *n* (%)	1 (4.2%)	0
Any solicited local AE, *n* (%)	14 (58.3%)	0
Erythema	3 (12.5%)	0
Swelling	1 (4.2%)	0
Pain/tenderness	12 (50.0%)	0
Any solicited systemic AE, *n* (%)	18 (75.0%)	5 (41.7%)
Fatigue	13 (54.2%)	2 (16.7%)
Headache	14 (58.3%)	2 (16.7%)
Nausea	4 (16.7%)	2 (16.7%)
Myalgia	11 (45.8%)	0
Fever	7 (29.2%)	0
Any solicited systemic AE related to vaccine, *n* (%)	18 (75.0%)	5 (41.7%)
Any unsolicited AE, *n* (%)	6 (25.0%)	1 (8.3%)
Vomiting	4 (16.7%)	0
Blood pressure increased	1 (4.2%)	0
Blood pressure systolic increased	1 (4.2%)	0
Dermatitis contact	1 (4.2%)	1 (8.3%)
Dry eye	1 (4.2%)	0
Any at least Grade 3 unsolicited AE, *n* (%)	0	0
Any unsolicited AE thought to be related to vaccine, *n* (%)	5 (20.8%)	0

*Note:* Adverse events were coded using MedDRA Version 23.1. Participants were counted only once for any given event, regardless of the number of times they experienced the event.

Abbreviations: AE = adverse event, *N* = total number of participants, *n* = number of participants in each category.

**FIGURE 1:**
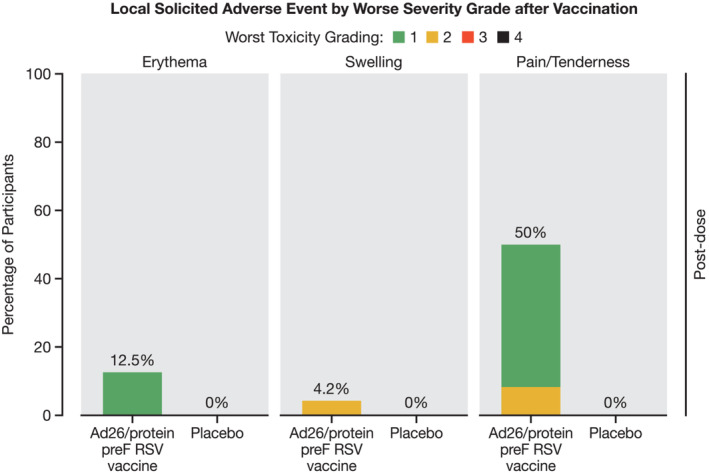
All local solicited adverse events by worst toxicity grade after vaccination. RSV = respiratory syncytial virus. *Note:* Percentage is calculated using the number of participants in the given phase and treatment group as the denominator.

Overall, solicited AEs were reported in 19/24 (79.2%) participants in the Ad26.RSV.preF/RSV preF protein intervention group and 5/12 (41.7%) participants in the placebo group. Solicited local AEs were reported only in the Ad26.RSV.preF/RSV preF protein intervention group (14/24 [58.3%] participants). The most frequently reported solicited local AE was pain/tenderness in 12/24 (50.0%) participants. In the Ad26.RSV.preF/RSV preF protein intervention group, the median duration of the most frequently reported solicited local AE (pain/tenderness) was 3.0 (range: 2–8) days, and its median time to onset was 2.0 (range: 1–2) days (Table S2 and Figure 1).

**FIGURE 2:**
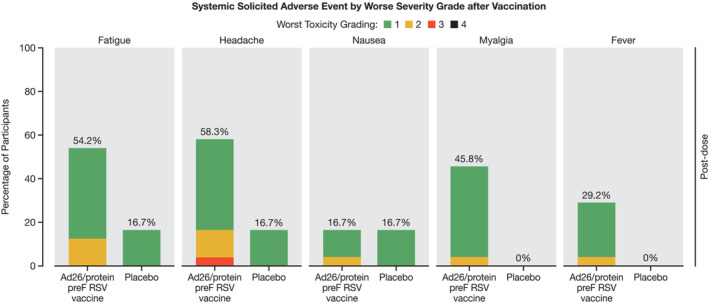
Systemic solicited adverse events by worst toxicity grade after vaccination. RSV = respiratory syncytial virus. Percentage is calculated using the number of participants in the given phase and treatment group as the denominator.

Solicited systemic AEs were reported in 18/24 (75.0%) participants and 5/12 (41.7%) participants in the Ad26.RSV.preF/RSV preF protein intervention group and placebo group, respectively. The most frequently reported solicited systemic AEs in the Ad26.RSV.preF/RSV preF protein intervention group were headache (14/24 [58.3%] participants), fatigue (13/24 [54.2%] participants), myalgia (11/24 [45.8%] participants), and fever (7/24 [29.2%] participants). The median duration and time to onset of the solicited systemic AEs in the Ad26.RSV.preF/RSV preF protein intervention group for nausea was 3 days, and for fatigue, headache, myalgia, and fever, it was typically 2 days (Table S3 and Figure 2).

Unsolicited AEs were reported in 6/24 (25.0%) participants in the Ad26.RSV.preF/RSV preF protein intervention group and 1/12 (8.3%) participants in the placebo group. Unsolicited AEs considered by the investigator to be related to vaccination were reported in 5/24 (20.8%) participants in the Ad26.RSV.preF/RSV preF protein intervention group and none in the placebo group. Overall, there were no noteworthy changes from baseline in any of the clinical chemistry or hematology laboratory parameters over time in any of the intervention groups. No clinically relevant changes from baseline or differences between the intervention groups were noted in vital sign measurements (Table S2).

### 3.2 Immunogenicity Findings

**FIGURE 3:**
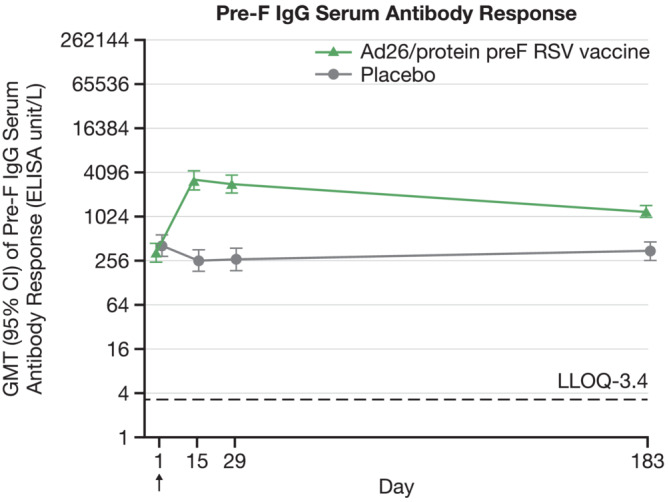
Pre‐F IgG serum antibody response (ELISA). Geometric mean titers with 95% CI are shown in the figure. CI = confidence interval, GMT = geometric mean titer, LLOQ = lower limit of quantification, RSV = respiratory syncytial virus.

At baseline, GMT of pre‐F IgG serum antibody was quantifiable in both intervention groups (GMT: 336 in Ad26/protein preF RSV vaccine and 418 in placebo groups). At Day 15, there was 9.6‐fold rise from baseline in GMT with pre‐F IgG serum antibody titer of 3220 in Ad26/protein preF RSV vaccine group. The pre‐F IgG serum antibody titer was 2893 at Day 29 and maintained above baseline up to Day 183 (GMT was 1212) in Ad26/protein preF RSV vaccine group (Table 3 and Figure 3).

**TABLE 3:** Pre‐F IgG serum antibody response.

	Ad26/protein preF RSV vaccine	Placebo
Analysis set: per‐protocol immunogenicity analysis set	24	12
Baseline		
*N*	24	12
Geometric mean (95% CI)	336 (248; 455)	418 (297; 587)
Day 15		
*N*	24	12
Geometric mean (95% CI)	3220 (2372; 4371)	259 (189; 357)
Geometric mean increase (95% CI)	9.6 (5.7; 16.1)	0.6 (0.5; 0.7)
Day 29		
*N*	24	12
Geometric mean (95% CI)	2893 (2184; 3833)	274 (192; 390)
Geometric mean increase (95% CI)	8.6 (5.4; 13.6)	0.7 (0.6; 0.8)
Day 183		
*N*	24	12
Geometric mean (95% CI)	1212 (991; 1483)	353 (265; 468)
Geometric mean increase (95% CI)	3.6 (2.7; 4.9)	0.8 (0.8; 0.9)

*Note:* Percentage is calculated using the number of participants with baseline and corresponding visit as the denominator.

Abbreviations: CI = confidence interval, *N* = number of participants with data, RSV = respiratory syncytial virus.

The titer of post‐F IgG serum antibody (GMT: 250 in Ad26/protein preF RSV vaccine and 296 in placebo group) and neutralizing antibodies to RSV‐A2 strain (GMT: 376 in Ad26/protein preF RSV vaccine and 410 in placebo group) was approximately similar between the intervention groups at baseline. At Day 15, there was 7.9‐fold rise in GMT with post‐F IgG serum antibody titer of 1980 in Ad26/protein preF RSV vaccine group. In Ad26/protein preF RSV vaccine group, the post‐F IgG serum antibody titers were 1759 at Day 29 (Table S4 and Figure S1).

**FIGURE 4:**
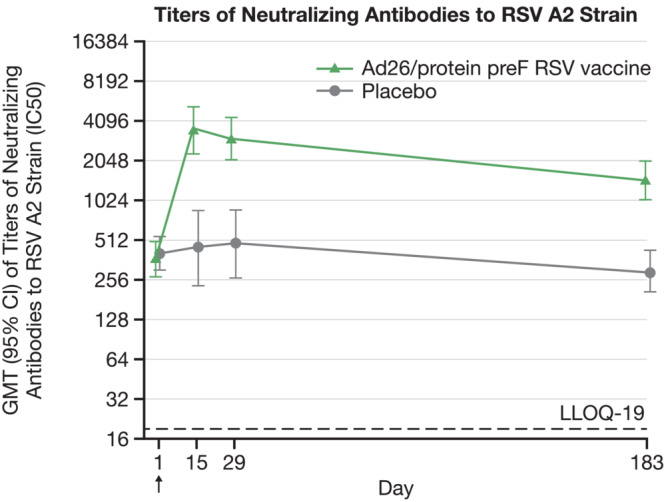
Titers of neutralizing antibodies to RSV‐A2 strain. Geometric mean titers with 95% CI are shown in the figure. GMT = geometric mean titer, LLOQ = lower limit of quantification, RSV = respiratory syncytial virus.

For neutralizing antibodies to RSV‐A2 strain, there was 9.3‐fold rise in GMT with neutralizing antibodies titer of 3497 in Ad26/protein preF RSV vaccine group at Day 15. In Ad26/protein preF RSV vaccine group, the GMT of neutralizing antibodies to RSV‐A2 strain were 3022 at Day 29 and maintained above baseline up to Day 183 (GMT was 1459) (Table 4 and Figure 4).

**TABLE 4:** Titers of neutralizing antibodies to RSV‐A2 strain.

	Ad26/protein preF RSV vaccine	Placebo
Analysis set: per‐protocol immunogenicity analysis set	24	12
Baseline		
*N*	24	12
Geometric mean (95% CI)	376 (278; 507)	410 (307; 549)
Day 15		
*N*	24	12
Geometric mean (95% CI)	3497 (2321; 5270)	455 (239; 864)
Geometric mean increase (95% CI)	9.3 (5.4; 15.9)	1.1 (0.7; 1.9)
Day 29		
*N*	24	12
Geometric mean (95% CI)	3022 (2087; 4375)	487 (271; 876)
Geometric mean increase (95% CI)	8 (4.9; 13.1)	1.2 (0.8; 1.8)
Day 183		
*N*	24	12
Geometric mean (95% CI)	1459 (1038; 2052)	297 (206; 430)
Geometric mean increase (95% CI)	3.9 (2.8; 5.5)	0.7 (0.6; 0.9)

*Note:* Percentage is calculated using the number of participants with baseline and corresponding visit as the denominator.

Abbreviations: CI = confidence interval, *N* = number of participants with data.

In addition, a 11.2‐fold rise from baseline in GMT of neutralizing antibodies to RSV‐B strain was observed at Day 15 (neutralizing antibodies titer of 24,459) in the Ad26/protein preF RSV vaccine group and was maintained (titer of 22,907) at Day 29 (Table S5 and Figure S2).

No increase from baseline in pre‐F IgG serum antibody titer, post‐F IgG serum antibody titer and neutralizing antibodies to RSV‐A2 or to RSV‐B was observed in the placebo over time.

## 4 Discussion

The results from this single‐center, randomized, placebo‐controlled, double‐blind, Phase 1 study presents the safety, reactogenicity, and immunogenicity evaluation of Ad26.RSV.preF/RSV preF protein vaccine candidate in Japanese adults aged ≥ 60 years. Although the required protective immune response is currently unknown, this vaccine strategy is considered to offer two advantages that can potentially counteract RSV infection, the induction of RSV‐specific neutralizing antibody response, and cell‐mediated immunity [18, 19, 20]. In the present study, the Ad26.RSV.preF/RSV preF protein vaccine candidate had an acceptable safety and tolerability profile in Japanese adults aged ≥ 60 years who were in stable health. No safety concerns associated with the vaccine were identified. There were no deaths, SAEs, or AEs leading to study discontinuation reported during the study. The reactogenicity was higher than placebo and in line with other studies [15, 21]. The frequency and intensity of solicited AEs were similar to those reported with Ad26‐vectored vaccines evaluated in other clinical trials [16, 22, 23, 24].

In older adult, the Ad26.RSV.preF/RSV preF protein vaccine induced RSV‐specific humoral immune responses, with an increase in RSV neutralizing antibody titers on Days 15 and 29 compared with baseline. GMTs of neutralizing antibodies to RSV‐A2 and of antibodies detected in a pre‐F ELISA peaked at Day 15 and were well maintained above baseline until Day 183. In addition to neutralizing antibody responses to the RSV‐A2 strain, neutralizing antibodies to the RSV‐B strain were detected, which has also been observed for other F‐based vaccines [24, 25]. These cross‐reactive responses suggest that a vaccine approach based on RSV‐A2 strain has the potential to provide protection against both RSV‐A and RSV‐B [26]. The data are encouraging, but in the absence of a definite correlate of protection, an efficacy study assessment is required in Japanese population. The immunogenicity results are in line with those described with other RSV vaccines based on the prefusion F protein [27, 28, 29, 30, 31, 32, 33]. In Phase 1/2 study, RSV preF protein formulations elicited robust neutralizing responses in older adults with RSV‐A (GM fold rises ranged from 4.8 to 11.6), and RSV‐B (GM fold rises ranged from 4.5 to 14.1) neutralizing GMTs increased at 1 month after vaccination [34]. In another Phase 1/2 randomized study, RSVPreF3 vaccines boosted humoral (RSVPreF3‐specific IgG [mean fold rise ranged from 7.2 to 12.8 on Day 31 and 5.5 to 9.3 on Day 91] and RSV‐A [mean fold rise ranged from 5.6 to 9.9 on Day 31 and 3.8 to 6.6 on Day 91] neutralizing antibody) responses, which increased in an antigen concentration‐dependent manner and were highest after Dose 1 [35].

Importantly, the pre‐F binding and RSV neutralizing titers remained elevated above baseline until at least Day 183. This durability could be an essential feature of a vaccine for the older adult population. Overall, Ad26.RSV.preF/RSV preF protein vaccine was well tolerated, had an acceptable safety profile, and was immunogenic after a single dose leading to commence a Phase 3 study in Japanese population.

## 5 Limitation

The study included a small number of participants to evaluate safety and immunogenicity of Ad26.RSV.preF/RSV preF protein vaccine and did not evaluate preventable efficacy against RSV infection.

## 6 Conclusions

The Ad26.RSV.preF/RSV preF protein vaccine was tolerable with no safety concerns and yielded an immunogenic response after single dose in Japanese adults aged ≥ 60 years who were in stable health.

## Author Contributions

**Takashi Eto:** data curation, investigation, writing–review and editing. **Yusuke Okubo:** formal analysis, writing–original draft, writing–review and editing. **Atsushi Momose:** formal analysis, writing–original draft, writing–review and editing. **Hiroshi Tamura:** conceptualization, formal analysis, methodology, writing–original draft, writing–review and editing. **Richuan Zheng:** conceptualization, formal analysis, methodology, writing–original draft, writing–review and editing. **Benoit Callendret:** conceptualization, formal analysis, methodology, writing–original draft, writing–review and editing. **Arangassery Rosemary Bastian:** conceptualization, formal analysis, methodology, writing–original draft, writing–review and editing. **Christy A. Comeaux:** conceptualization, formal analysis, methodology, writing–original draft, writing–review and editing.

## Conflicts of Interest

Y.O., A.M., H.T., and R.Z. are employees of Janssen Pharmaceutical K.K. B.C., A.R.B., and C.A.C. are employees of Janssen Pharmaceuticals. T.E. declares no conflicts of interest.

### Peer Review

The peer review history for this article is available at https://www.webofscience.com/api/gateway/wos/peer‐review/10.1111/irv.13336.

## Ethics Statement

The investigatory review board at each study site approved the study protocol.

## Consent

All participants provided their informed consent in writing prior to enrolling in the study.

## Supporting information


**Figure S1.** Post‐F IgG Serum Antibody Response (ELISA)

**Figure S2.** Titers of Neutralizing Antibodies to RSV B Strain

**Table S1.** Patient Disposition
**Table S2.** Overall Summary of Local Solicited Adverse Events by Grade
**Table S3.** Overall Summary of Systemic Solicited Adverse Events by Grade
**Table S4.** Post‐F IgG Serum Antibody Response
**Table S5.** Titers of Neutralizing Antibodies to RSV B Strain

## Data Availability

The data sharing policy of Janssen Pharmaceutical Companies of Johnson & Johnson is available at https://www.janssen.com/clinical‐trials/transparency. The data supporting the findings of this study and may be obtained from the authors upon reasonable request.
