# Supplementary material for: A Randomized, Double‐Blind, Placebo‐Controlled, Phase 1 Study to Evaluate the Safety, Reactogenicity, and Immunogenicity of Single Vaccination of Ad26.RSV.preF‐Based Regimen in Japanese Adults Aged 60 Years and Older
Source: Influenza Other Respir Viruses. 2024 Jun 16;18(6):e13336. doi: 10.1111/irv.13336 (PMC11180550; doi:10.1111/irv.13336)
Supplement: Supplementary file 3 — Table S1. Patient Disposition Table S2. Overall Summary of Local Solicited Adverse Events by Grade Table S3. Overall Summary of Systemic Solicited Adverse Events by Grade Table S4. Post‐F IgG Serum Antibody Response Table S5. Titers of Neutralizing Antibodies to RSV B Strain [file IRV-18-e13336-s003.docx]

# SUPPLEMENTAL MATERIAL

**Supplementary Table 1. Patient Disposition**

|  | **Ad26/protein preF RSV vaccine** | **Placebo** | **All Participants** |
| --- | --- | --- | --- |
| Analysis set: Full Analysis Set | 24 | 12 | 36 |
|  |  |  |  |
|  |  |  |  |
| Participants ongoing | 0 | 0 | 0 |
| Completed study participation | 24 (100.0%) | 12 (100.0%) | 36 (100.0%) |
| Discontinued study participation | 0 | 0 | 0 |
| Reason for discontinuation |  |  |  |
| No data to report | - | - | - |
| RSV = respiratory syncytial virus  The denominator for the percentage calculation is the full analysis set. | | | |

**Supplementary Table 2. Overall Summary of Local Solicited Adverse Events by Grade**

|  | **Ad26/protein preF RSV vaccine** | **Placebo** |
| --- | --- | --- |
| Analysis set: Full Analysis Set | 24 | 12 |
|  |  |  |
|  |  |  |
| Post-dose | 24 | 12 |
|  |  |  |
| Participants with 1 or more solicited local AEs |  |  |
| Any | 14 (58.3%) | 0 |
| Grade 1 | 11 (45.8%) | 0 |
| Grade 2 | 3 (12.5%) | 0 |
|  |  |  |
| Erythema |  |  |
| Any | 3 (12.5%) | 0 |
| Grade 1 | 3 (12.5%) | 0 |
|  |  |  |
| Swelling |  |  |
| Any | 1 (4.2%) | 0 |
| Grade 2 | 1 (4.2%) | 0 |
|  |  |  |
| Pain/Tenderness |  |  |
| Any | 12 (50.0%) | 0 |
| Grade 1 | 10 (41.7%) | 0 |
| Grade 2 | 2 (8.3%) | 0 |
| AE = adverse event, RSV = respiratory syncytial virus Percentage is calculated using the number of participants in the given Phase and treatment group as the denominator. Note: Participants are counted only once for any given event, regardless of the number of times they actually experienced the event. The event experienced by the participant with the worst toxicity grade is used. If a participant has missing toxicity grade for a specific adverse event, the participant is counted in the ’Any’ row for that adverse event. | | |

**Supplementary Table 3. Overall Summary of Systemic Solicited Adverse Events by Grade**

|  | **Ad26/protein preF RSV vaccine** | **Placebo** |
| --- | --- | --- |
| Analysis set: Full Analysis Set | 24 | 12 |
|  |  |  |
| Post-dose | 24 | 12 |
|  |  |  |
| Participants with 1 or more solicited systemic AEs |  |  |
| Any | 18 (75.0%) | 5 (41.7%) |
| Grade 1 | 13 (54.2%) | 5 (41.7%) |
| Grade 2 | 4 (16.7%) | 0 |
| Grade 3 | 1 (4.2%) | 0 |
|  |  |  |
| Fatigue |  |  |
| Any | 13 (54.2%) | 2 (16.7%) |
| Grade 1 | 10 (41.7%) | 2 (16.7%) |
| Grade 2 | 3 (12.5%) | 0 |
|  |  |  |
| Headache |  |  |
| Any | 14 (58.3%) | 2 (16.7%) |
| Grade 1 | 10 (41.7%) | 2 (16.7%) |
| Grade 2 | 3 (12.5%) | 0 |
| Grade 3 | 1 (4.2%) | 0 |
|  |  |  |
| Nausea |  |  |
| Any | 4 (16.7%) | 2 (16.7%) |
| Grade 1 | 3 (12.5%) | 2 (16.7%) |
| Grade 2 | 1 (4.2%) | 0 |
|  |  |  |
| Myalgia |  |  |
| Any | 11 (45.8%) | 0 |
| Grade 1 | 10 (41.7%) | 0 |
| Grade 2 | 1 (4.2%) | 0 |
|  |  |  |
| Fever |  |  |
| Any | 7 (29.2%) | 0 |
| Grade 1 | 6 (25.0%) | 0 |
| Grade 2 | 1 (4.2%) | 0 |
| AE = adverse event, RSV = respiratory syncytial virus Percentage is calculated using the number of participants in the given Phase and treatment group as the denominator. Note: Participants are counted only once for any given event, regardless of the number of times they actually experienced the event. The event experienced by the participant with the worst toxicity grade is used. If a participant has missing toxicity grade for a specific adverse event, the participant is counted in the ’Any’ row for that adverse event. | | |

**Supplementary Table 4. Post-F IgG Serum Antibody Response**

|  | **Ad26/protein preF RSV vaccine** | **Placebo** |
| --- | --- | --- |
|  |  |  |
| **Analysis set: Per Protocol Immunogenicity Analysis Set** | 24 | 12 |
|  |  |  |
| **Baseline** |  |  |
| N | 24 | 12 |
| Geometric mean (95% CI) | 250 (188; 332) | 296 (210; 418) |
|  |  |  |
| **Day 15** |  |  |
| N | 24 | 12 |
| Geometric mean (95% CI) | 1980 (1464; 2678) | 228 (167; 312) |
| Geometric mean increase (95% CI) | 7.9 (5; 12.5) | 0.8 (0.7; 0.9) |
|  |  |  |
| **Day 29** |  |  |
| N | 24 | 12 |
| Geometric mean (95% CI) | 1759 (1333; 2321) | 241 (177; 330) |
| Geometric mean increase (95% CI) | 7 (4.8; 10.4) | 0.8 (0.7; 0.9) |
| CI = confidence interval, N = number of participants with data, RSV = respiratory syncytial virus Percentage is calculated using the number of participants with baseline and corresponding visit as the denominator. | | |

**Supplementary Table 5. Titers of Neutralizing Antibodies to RSV B Strain**

|  | **Ad26/protein preF RSV vaccine** | **Placebo** |
| --- | --- | --- |
|  |  |  |
| **Analysis set: Per Protocol Immunogenicity Analysis Set** | 24 | 12 |
|  |  |  |
| **Baseline** |  |  |
| N | 23 | 12 |
| Geometric mean (95% CI) | 2237 (1667; 3001) | 3152 (2406; 4129) |
|  |  |  |
| **Day 15** |  |  |
| N | 23 | 12 |
| Geometric mean (95% CI) | 24459 (17580; 34029) | 2998 (2255; 3985) |
| Geometric mean increase (95% CI) | 11.2 (6.7; 18.8) | 1 (0.9; 1.1) |
|  |  |  |
| **Day 29** |  |  |
| N | 9 | 4 |
| Geometric mean (95% CI) | 22907 (13649; 38445) | 2253 (1947; 2608) |
| Geometric mean increase (95% CI) | 11.6 (5.9; 23) | 1 (0.8; 1.2) |
| N = number of participants with data, CI = confidence interval, LLOQ = lower limit of quantification. Percentage is calculated using the number of participants with baseline and corresponding visit as the denominator. | | |

**SUPPLEMENTARY FIGURE LEGENDS**

**Supplementary Figure 1. Post-F IgG Serum Antibody Response (ELISA)**

Geometric mean titers with 95% CI are shown in the figure.

GMT = geometric mean titer, LLOQ = lower limit of quantification; RSV: respiratory syncytial virus

**Supplementary Figure 2. Titers of Neutralizing Antibodies to RSV B Strain**

Geometric mean titers with 95% CI are shown in the figure.

GMT = geometric mean titer, LLOQ = lower limit of quantification.; RSV = respiratory syncytial virus
